# Screened selection design for randomised phase II oncology trials: an example in chronic lymphocytic leukaemia

**DOI:** 10.1186/1745-6215-12-S1-A91

**Published:** 2011-12-13

**Authors:** Christina Yap, Lucinda Billingham, Andrew Pettitt

**Affiliations:** 1 MRC Midland Hub for Trials Methodology Research, University of Birmingham, Birmingham, B15 2TT, UK; 2 Cancer Research UK Clinical Trials Unit, University of Birmingham, Birmingham, B15 2TT, UK; 3 Molecular and Clinical Cancer Medicine, University of Liverpool, Liverpool, L69 3GA, UK

## Background

There is a growing number of potential new treatment regimens for chronic lymphocytic leukaemia (CLL). As there is limited number of patients, it is important that statistical methodologies in Phase II trials efficiently select promising regimens for subsequent evaluation in a confirmatory, larger-scale Phase III trial.

## Methods

In this study, we propose the screened selection design, which combines two conventional Phase II trial designs to provide a practical multi-stage approach for evaluating the efficacy of two experimental arms in high-risk CLL patients. Our aim is to select the most promising regimen in terms of effectiveness to be recommended for further testing. The proposed Phase II randomised design is divided into two different segments. In the first segment, patients are randomised equally into two experimental arms. By applying Simon’s two-stage design [1] in each of the two parallel experimental arms, this allows for initial determination of efficacy and early stopping for futility in any of the arms. If there are an insufficient number of responses in the first stage, recruitment will not continue for that particular arm. Otherwise, the study proceeds to stage 2 to randomize further patients to each arm. The second segment of the study involves the play-the-winner selection strategy as proposed by Simon, Wittes and Ellenberg [2], which only applies if results from both arms are found to be positive. Our proposed design allows the treatment arm with the highest response rates to be recommended only if the efficacy rate is greater than a specified clinically-relevant value. The number of subjects required for each treatment arm in the first segment is selected to be as close as possible to the number required for the selection strategy in the second segment according to pre-specified error rates. The operating characteristics of the trial design are explored via a simulation study.

## Results

The proposed approach has the advantage of substantial reduction in the probability of incorrectly selecting an ineffective arm whose rates are not clinically significant or when no true difference exists between the arms. The only compromise is a slight reduction in the probability of correctly selecting an effective treatment arm if one exists. This approach is comparable to the Bayesian Selection Strategy proposed by Estey and Thall [3].

## Conclusions

The proposed approach provides an easy to implement Phase II design to select an effective and most promising treatment regimen for further testing in Phase III.
